# Cerebral Palsy: A Dental Update

**DOI:** 10.5005/jp-journals-10005-1247

**Published:** 2014-08-29

**Authors:** Nidhi Sehrawat, Mohita Marwaha, Kalpana Bansal, Radhika Chopra

**Affiliations:** Postgraduate Student, Department of Pedodontics, SGT Dental College, Gurgaon Haryana, India; Reader, Department of Pedodontics and Preventive Dentistry, SGT Dental College and Research Institute, Gurgaon, Haryana India; Professor and Head, Department of Pedodontics and Preventive Dentistry, SGT Dental College, Gurgaon, Haryana, India; Reader, Department of Pedodontics, ITS Dental College, Ghaziabad Uttar Pradesh, India

**Keywords:** Cerebral palsy, Dental considerations, Management

## Abstract

Special and medically compromised patients present a unique population that challenges the dentist’s skill and knowledge. Providing oral care to people with cerebral palsy (CP) requires adaptation of the skills we use everyday. In fact, most people with mild or moderate forms of CP can be treated successfully in the general practice setting. This article is to review various dental considerations and management of a CP patient.

**How to cite this article:** Sehrawat N, Marwaha M, Bansal K, Chopra R. Cerebral Palsy: A Dental Update. Int J Clin Pediatr Dent 2014;7(2):109-118.

## INTRODUCTION

Special and medically compromised patients present a unique population that challenges the dentist’s skill and knowledge. People with disabilities often need extra help to achieve and maintain good oral health.^1^

Census (2001) has revealed that over 21 million people in India^2^ are suffering from one or the other kind of disability. One of the most common forms of neuromuscular disabilities affecting children is cerebral palsy (CP), the worldwide incidence being 2 to 2.5 per 1000 live births.^3^

Cerebral palsy is a condition caused by damage to the brain, usually occurring before, during or shortly after birth. ‘Cerebral’ refers to the brain and ‘palsy’ refers to a disorder of movement or posture. Cerebral palsy is a central nervous system (CNS) disorder of movement, coordination, and posture, refecting a nonprogressive abnormality or insult to the immature brain.^4^ Cerebral palsy is neither progressive nor communicable. It is also not curable, although education, therapy and applied technology can help people with CP lead productive lives.

Providing oral care to people with cerebral palsy requires adaptation of the skills we use everyday. In fact, most people with mild or moderate forms of CP can be treated successfully in the general practice setting.

## HISTORY AND DEFINITION

The history of CP is a long one, dating back to ancient Egypt. There are at least two drawings of individuals from the fifth century BC with what is recognized today as spastic CP. William John Little is credited with the first description of CP in 1843. He coined the term ‘cerebral palsy’ in 1889.^5^

American Academy of Cerebral Palsy (AACP) was formed in 1947. An international workshop on definition and classification of CP was held in Bethesda, Maryland, July 11 to 13, 2004, because of a perceived need to revisit the definition and classification of CP.^5^

Cerebral palsy describes a group of permanent disorders of the development of movement and posture, causing activity limitations, that are attributed to non-progressive disturbances that occurred in the developing fetal or infant brain. The motor disorders of CP are often accompanied by disturbances of sensation, perception, cognition, communication, behavior, by epilepsy and by secondary musculoskeletal problems.^6^

## ETIOLOGY

The exact etiology can be identified only in 40 to 50% of the cases.^1^ Approximately, 30% of cases have none of the known risk factors.^7^ The condition or risk factors associated with CP can be broken down into those occurring in the prenatal, perinatal or postnatal time period.^8^ It has been estimated that up to 70 to 80% of CP cases can be attributed to prenatal factors with birth asphyxia responsible for a small number (approximately 10%) and remainder due to identifable postnatal conditions.^9^

The prenatal risk factors associated with CP included hypoxia, genetic and metabolic disorders, multiple gestation, intrauterine infections, thrombophilic disorders, teratogenic exposure, chorioamnionitis, maternal fever, exposure to toxins, malformations of brain structures, intrauterine growth restriction, abdominal trauma and vascular insults.

The perinatal risk factors associated with CP include asphyxia, premature birth (<32 weeks or <2500 gm), blood incompatibility, infection, abnormal fetal presentation, placental abruption and instrument delivery.

The postnatal risk factors associated with CP include asphyxia, seizures in postnatal period, cerebral infarction, hyperbilirubinemia, sepsis, respiratory distress syndrome, chronic lung disease, meningitis, postnatal steroids, intraventricular hemorrhage, periventricular leukomalacia, shaken baby syndrome and head injury.

## CLASSIFICATION

Minear and the nomenclature and classification committee of the American Academy of CP in 1956 presented a set of potential classification schemes that have remained pertinent over the years.^10^ This early classification system included broad clinical symptoms with categories for physiology (the nature of the motor abnormality), topographic, etiology, neuroanatomic features, supplemental (associated) conditions, functional capacity (severity) and therapeutic requirements.

**Figs 1A to E:**
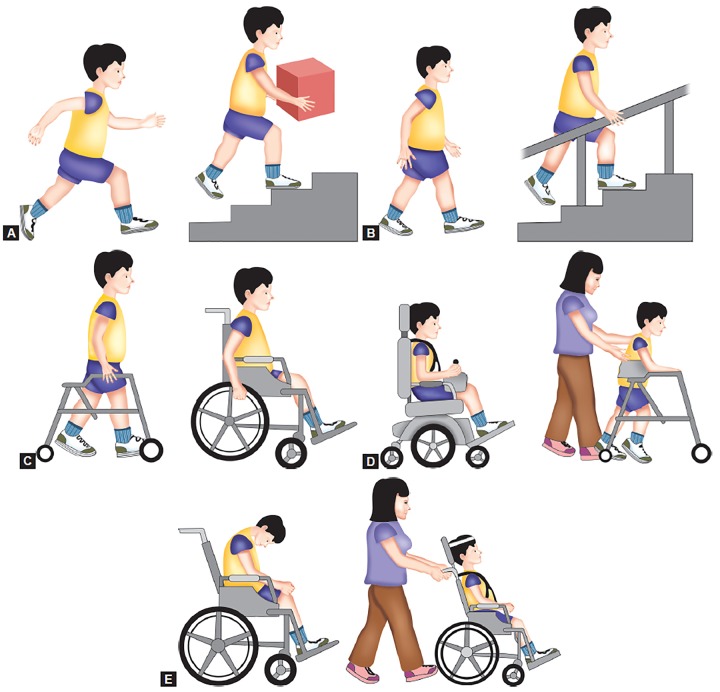
Gross motor function classification system (GMFCS): (A) Level I – walks without limitations, (B) Level II – walks with limitations, (C) Level III – walks using a hand-held mobility device, (D) Level IV – self-mobility with limitations may use powered mobility and (E) Level V – transported in a manual wheelchair

In 1997, a hallmark paper was published by Palisano et al^11^ that provided a new classification system for gross motor function in children with CP, the gross motor function classification system (GMFCS). This original GMFCS had some limitations. These limitations included an upper age limit of 12 years (before adolescence) and the necessity of using a single rating to describe a child’s ambulatory performance across different terrains and distances, resulting in a tendency of parents and therapists to rate a child based on their best capacity rather than their typical performance when forced by the rating scale to choose a single category. These issues were considered and addressed in an updated version of the scale that was given in 2007.^13^ The GMFCS-expanded and revised (GMFCS-ER) included children up to 18 years of age.^12^ It classifes gross motor function on a five-point ordinal scale, with descriptions of skills provided for five age groups: less than 2 years of age, 2 to 4 years of age, 4 to 6 years of age, 6 to 12 years of age and finally 12 to 18 years of age. In general, the levels are given in Figures 1A to E.

The international classification of functioning (ICF), disability and health was developed by the World Health Organization (WHO) and endorsed by the World Health Assembly in May 2001.^14^ ICF conceptualizes a person’s level of functioning as a dynamic interaction between his or her health conditions, environmental and personal factors. The ICF describes disability as dysfunction at one or more of these levels: impairment of body structure (organs or limbs) or functions (physiologic or psychological), limitations in activities (execution of tasks or actions by the individual) and restriction of participation (involvement in life situations).^12^

**Figs 2A to F:**
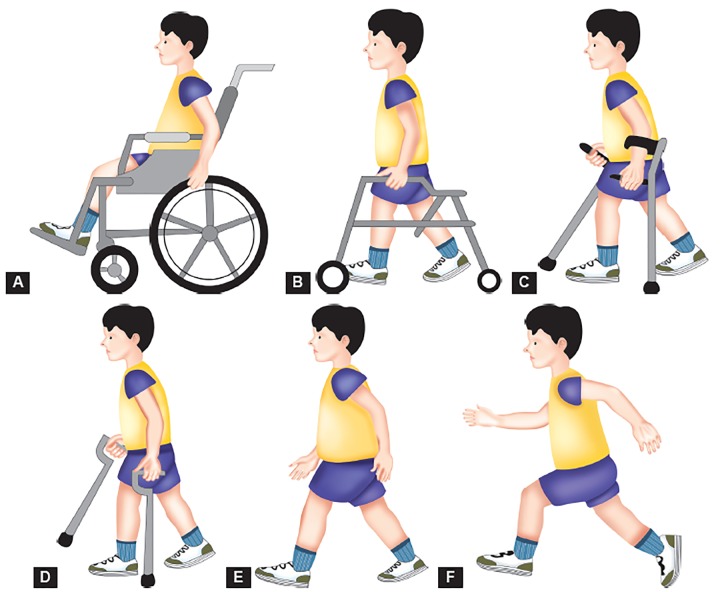
Functional mobility scale (FMS): (A) Rating 1– uses wheelchair, (B) Rating 2 – uses walker or frame, (C) Rating 3 –uses crutches, (D) Rating 4 – uses sticks (canes), (E) Rating 5 – independent on level surfaces and (F) Rating 6 – independent on all surfaces

In 2004,Graham et al^15^ designed the functional mobility scale (FMS) as a measure of ambulatory performance in children with CP. Functional mobility scale is the only existing functional scale that accounts for the fact that children may demonstrate different ambulatory abilities and use different assistive devices to walk various distances. Functional mobility scale is administered via parent/patient interview and categorizes the assistance needed (none, canes, crutches, walker, wheelchair) for a child to walk three distances (5, 50 and 500 yards, or 5, 50 and 500 m). The distances are not specifically measured, but used as estimates to represent household, school and community ambulation. Ratings are given for each distance category as given in Figures 2A to F. Rating C: Child crawls the designated distance; Rating N: Child is unable to move through a given distance. A child who ambulates independently for all the distances and on all types of surfaces would be given a Rating of 6, 6 and 6. The FMS specifically addresses ambulation and, therefore, is not intended to substitute for the GMFCS, which assesses mobility on a more general level. The FMS should be used as a companion rating scale to the GMFCS.^12^

In 2006, the manual ability classification system (MACS)^16^ was designed to describe the upper-extremity performance in activities of daily living for children with CP. The MACS is also designed as a five-category scale and the levels include the following:

- *Level I*: Handles objects easily and successfully.
- *Level II*: Handles most objects but with somewhat reduced quality or speed of achievement.
- *Level III*: Handles objects with difficulty; needs help to prepare or modify activities.
- *Level IV*: Handles a limited selection of easily managed objects in adapted situations.
- *Level V*: Does not handle objects and has severely limited ability to perform even simple actions.

In 2011, communication function classification system (CFCS) was given by Hidecker MJC, Paneth N et al.^17^ The purpose of the CFCS is to classify the everyday communication performance of an individual with CP into one of the five levels. The five levels include the following:^18^

- *Level I*: Effective sender and receiver with unfamiliar and familiar partners.
- *Level II*: Effective, but slower-paced sender and/or receiver with unfamiliar and familiar partners.
- *Level III*: Effective sender and effective receiver with familiar partners.
- *Level IV*: Inconsistent sender and/or receiver with familiar partners.
- *Level V*: Seldom effective sender and receiver with familiar partners.

## DIAGNOSIS

The diagnosis of CP is not always straightforward, but an early diagnosis is important in terms of optimizing therapeutic interventions. Clinical observations and parental report are the initial stages in formulating this diagnosis. Children who are severely affected, or who have a known risk factor, often are diagnosed at an earlier age than those who are affected more mildly.^9^

It is not possible to diagnose CP in infants less than 6 months except in very severe cases.^3^ The natal history can often raise suspicions and merits closer monitoring. The major signs that collectively can lead to a CP diagnosis are delayed motor milestones, abnormal neurological examination, persistence of primitive refexes and abnormal postural actions.^8^

In many cases, a diagnosis of CP may not be possible till 12 months. Repeated examinations and observations over a period of time may be required in mild cases before a firm diagnosis can be made.^3^ The diagnosis often is easier if there is known brain damage documented by cranial ultrasound, computed tomography (CT) or magnetic resonance imaging (MRI).^8^

Genetic and metabolic tests are carried out if there is evidence of deterioration or metabolic compensation, family history of childhood neurological disorder associated with CP. Tests to rule out coagulopathy in children with stroke is necessary. Complete evaluation of a child with CP should include an assessment of associated deficits like vision, speech and hearing, sensory profile, oromotor evaluation, epilepsy and cognitive functioning. Orthopedic evaluation is must as muscle imbalance and spasticity cause subluxation/ dislocation of the hips, equinus deformities, contractures and scoliosis.^3^

## DENTAL MANIFESTATIONS

### Malocclusion

Prevalence rate of malocclusion has been reported between 59 and 92%,^9,19^ with vast majority of malocclusion classified as Angles Class II^9,19-22^ with increased overjet and overbite.^23,24,26^ The main risk factors associated with the severity of malocclusion were CP, mouth breathing, lip incompetence and long face.^24^ Also, they have a three-fold greater chance of developing anterior open bite.^25^ Distribution of open bite was found to decrease with increased age.^23^ Spastic patients presented with an increased incidence of open bite.^21^ High rate of Class II malocclusion and anterior open bite can be attributed to hypotonia of the orofacial muscles with resultant forward tongue posture, a poor swallow refex and frequent mouth breathing.^9^

### Traumatic Dental Injuries

Individuals with CP have a high prevalence of Class II malocclusion with prominent maxillary incisors, incompetent lips, difficulty in ambulation and increased incidence of seizures, all these predispose the individual to dental trauma.^9,26^ Holan et al^26^ found greatly increased incidence of dental trauma in CP population (57%). There are other studies that found the prevalence to be lesser (9.2-20%).^27-31^ Fracture of enamel and dentine was the most common type of injuries.^26,28,31^ Some studies suggested that the prevalence of traumatic dental injuries in individuals presenting CP and attending rehabilitation treatment was similar compared with nondisabled individuals, but they received less treatment.^31^

### Bruxism

Bruxism, the habitual grinding of teeth is a common occurrence in people with C P. In extreme cases, bruxism leads to tooth abrasion and fat biting surfaces.^27^ A high prevalence of bruxism in individuals who have CP has been reported in several articles.^32,33^ Ortega et al^32^ found that along with bruxism there were habits like pacifer-sucking, fingersucking, habit of biting objects and tongue interpositioning. Minear WL^10^ have hypothesized that bruxism habits in these population are related to problems with dopamine function and not regulated by local factors, such as malocclusion. It was shown that abnormal dental wear is more closely related to a low level of mental development that to the degree of severity of CP. The absence of proprioception in the periodontium is discussed as a possible cause of bruxism.^33^

### Dental Caries

Dental caries constitutes a multifactor disease in which different biological, economic, cultural, environmental and social factors interact^34^ the incidence of caries among children and adolescents who have CP is high, also the factors associated with the incidence of caries were similar to those affecting the population at large.^35^ Dietary consistency and oromotor function were statistically significant influence on the DMF index. Values were measured for individuals who were severely impaired and also the younger as well as for those receiving liquid diets. Early rehabilitation, intervention and prevention are important for these individuals.^36^

### Sialorrhea

Drooling of saliva appears to be the consequence of a dysfunction in the coordination of the swallowing mechanism, resulting in excess pooling of saliva in the anterior portion of the oral cavity and the unintentional loss of saliva from the mouth. Drooling can produce significant negative effects on physical health and quality of life, especially in patients with chronic neurological disabilities.^37^ Prevalence rates of sialorrhea in children who have CP are reported range from 10 to 58%.^38,39^

### Oral Hygiene

Poor oral hygiene is frequently cited as a problem affecting the oral health status of individuals who have CP.^40-42^ Santos MT et al^41^ concluded that the more severe the neurological damage is, the more frequent is the presence of the biting refex and consequently, the higher is the risk of oral diseases in this population due to the difficulty to perform an adequate oral hygiene.

### Dental Erosion

Most of the affected teeth observed in the CP group were upper molars (54%), lower molars (58%) and upper incisors (54%). It was concluded that dental erosion is common in CP patients, and dentists should be alert to early signs of dental erosion in cerebral palsy patients and provide appropriate preventive therapy and referral for diagnosis and treatment of gastroesophageal reflux disease to avoid irreversible damage to the dentition.^43^

### Enamel Defects

Most of the enamel defects were located symmetrically in the primary incisors and first molars. Around 42.4% of children with enamel defects were born prematurely (< 37 weeks) whereas only 23.2% of them were born at normal gestational age. No statistically significant difference in the prevalence of enamel defects was found in relation to birth weight (p > 0.05). It was concluded that a high prevalence of developmental enamel defects were found among the children with CP. The prevalence of defects varied with the tooth type and was associated with gestational age of the children.^44^

### TMJ Disorders

The presence of CP, male gender, severity of the malocclusion, mouth breathing and mixed dentition were identified as risk indicators for signs and symptoms of temporomandibular disorders. It was concluded that children with CP had a significantly greater chance of developing signs and symptoms of temporomandibular disorders.^45^

## DENTAL MANAGEMENT

### Dental Access

**Fig. 3:**
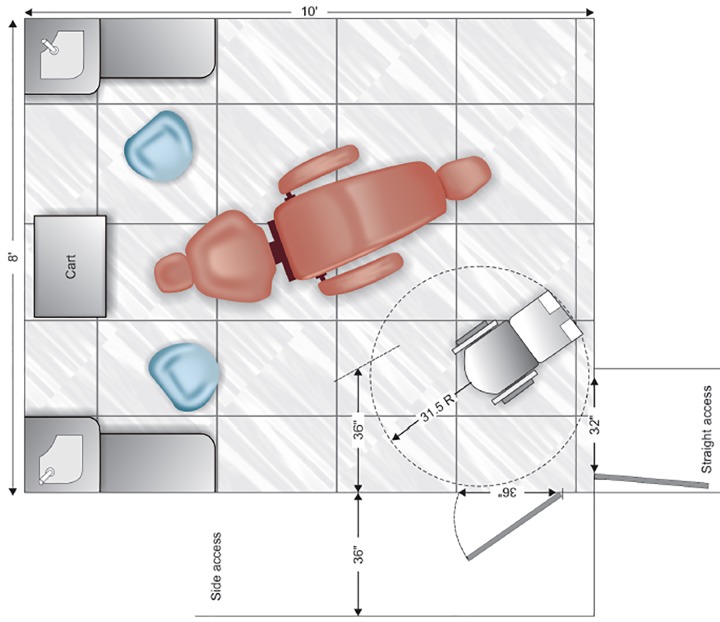
An accessible dental operatory foor plan designed for either a straight or side access doorway

Table 1 lists the common minimum requirements needed to gain access. In the dental suite where foor circulation space is at a premium, aisle passage in the operatory area should be planned with the dimensions shown in Figure 3.^46^

When seeing one of these children for dental examination or treatment, the dentist must bear in mind the problems that may lead to adjustment of his approach. These are:

1. Apprehension: Many of these children are not used to meet strangers.
2. Difficulty of communication: If there is an auditory, visual or speech defect, chairside communication must be modified accordingly.
3. Low intelligence: This can contribute to difficulty of cooperation.
4. Poor concentration: This may be an inherent aspect of the cerebral dysfunction, trivial things distracting the attention.
5. Convulsions: These are not common in the dental surgery as the child will be receiving drugs to control such episodes.
6. Posture: Ataxic patients need to have the dental chair tipped well back to give stability and support, while the spastic and athetoid may need more manual support and control in the chair.
7. Ability to cooperate: If the patient can sit in the chair and open his/her mouth, he/she can be treated as a normal patient. Those with less physical control need further help. Confidence and relaxation can overcome the problem in some.^47^

**Table 1:** **Table 1:** Accessibility guidelines

*External/internal building features*		*Gradient*		*Length*		*Width*		*Surface, other specifics*	
Parking space		1:50 max slope		Standard		Auto: 96 inches Van: 144 inches		Non-skid, paved, sign-posted, adjacent to walkway	
Walkway		1:12 max slope		Not applicable		36 inches		Non-skid, no obstructions, overhangs, smooth	
Passenger loading zone		Flat		20 feet		60 inches		Same as above	
Curb ramps		1:12 max slope				36 inches		Non-skid, side fair <1:10 slope	
Door		5 foot entrance and exit platform area		Standard		32 inches minimum; preferably 36 inches		Away from prevailing winds, lever with 10 lb pull, auto-assisted door available, kick plate	
Interior ramp		1:20 max slope		72 inch minimum length if rise >6 inches		36 inches		Non-skid, handrails	
Wheelchair lift		Bilevel		8 foot max drop		36 × 48 inches		Non-skid, dependent on specific chair	
Corridor				Standard		48 inches/64 inches		New facility, no obstacles	
Flooring		Flat, firm carpet		Not applicable		½ inch maximum thickness		No doormats, level thresholds	
Signs		Braille, raised letters		Above 5 feet		Readable		Near latch of office door	
Waiting room		Flat		Standard		36 inches aisle one cleared area: 36 *×* 52 inches		No carpet pad, well insulated, minimum low-frequency background noise	
Restrooms		Flat				32 inches stall minute, preferably 36 inches		Non-skid, magnetic catch door	
Public telephone		No higher than 4 feet		3 feet above foor		26 inches clearance		Phone directory near phone, adjustable volume control	
Elevator		Flat				54 *×* 68 inches		Non-skid, call and control box 48 inches high, including braille or incised letters	
Operatory		Flat (8 × 10 feet)		Standard		32 to 36 inches door		Non-skid, rotating or movable chair, drill and suction	

*First dental visit*: By scheduling the patient at a designated time (early in the day) and allowing sufficient time to talk with the parents (or the guardian) and the patient before initiating any dental care, a practitioner can establish an excellent relationship with them.^46^ The first visit or visits must be used primarily for the dentist and patient to become acquainted with each other and for the establishment of mutual confidence.^47^

*Radiographic examination*: Occasionally, assistance from the parent and dental auxiliaries and the use of immobilization devices may be necessary to obtain the films. An 18" (46 cm) length of foss is attached through a hole made in the tab, to facilitate retrieval of the film if it falls toward the pharynx.^47^

*Patient positioning*: The dental chair must be adjusted carefully and many of these patients are best treated with the chair tipped well back to give a position of security, especially to those with ataxia. The spastic with fairly severe head and neck involvement will need even more control and support and can be seated on the knee of the parent or an assistant, leaning back against the right shoulder.^47^

The assistive stabilization and postural maintenance can be achieved through the following techniques:^48^

1. Head position maintained in the midline by one of the dental staff over a head support (position device) located at the occipital level.
2. Maintenance of bent and juxtaposed upper members in the midline, with the help of Velcro straps.
3. Maintenance of bent lower members decreasing the hip angle to 120° in relation to the trunk using soft foam rolls as positioning devices, such as for support under the knees.
4. Maintenance of an open mouth with the use of mouth props.

In the spastic, sudden action may precipitate a spasm of the muscles, so the proposed action should be explained and the child encouraged to relax first. The mouth should then be opened with slow pressure by the dentist, but at no time should the fingers be allowed to come between the patient’s teeth in case of clenching. A finger guard may be used and a simple one is a steel thimble with a cord or chain attached through a hole drilled near the edge to prevent loss in the mouth. It is advisable to use a steel mirror which will not shatter. Also, great care must be taken where using sharp instruments to avoid damage to soft tissues.^47^

For conservation, a mouth prop is usually essential but it must be firmly placed and not left in for any considerable time as the child’s muscles tire quickly and frequent periods of rest are important. There is no reason why local anesthesia should not be used provided it can be given safely. A waterspray and some type of suction device are essential as the patient cannot rinse satisfactorily; a tongue retractor can be of great value. When placing the filling, it may be difficult to keep a dry field, but this may be assisted by the use of a rubber dam clamp without the rubber dam, to hold cotton-wool rolls in place, and has the advantage of being quick to fit and to remove. It should only be used in conjunction with a mouth prop.

Appliances, either orthodontic or prosthetic, are advisable only in those cases where the disability is slight or there is no danger of breakage and should be robust. Any treatment plan must be simple and within the limits of toleration of the child.^47^

*Home oral health practice*: Home dental care should begin in infancy; the dentist should teach the parents to gently cleanse the incisors daily with a soft cloth or an infant toothbrush. For older children who are unwilling or physically unable to cooperate, the dentist should teach the parent or guardian correct toothbrushing techniques that safely restrain the child when necessary. Some of the positions most commonly used for children requiring oral care assistance are as follows:

1. The standing or sitting child is placed in front of the adult so that the adult can cradle the child’s head with one hand while using the other hand to brush the teeth.
2. The child reclines on a sofa or bed with the head angled backward on the parent’s lap. Again, the child’s head is stabilized with one hand, while the teeth are brushed with the other hand.
3. The parents face each other with their knees touching. The child’s buttocks are placed on one parent’s lap, with the child facing that parent, while the child’s head and shoulders lie on the other parent’s knees; this allows the first parent to brush the teeth.
4. The extremely difficult patient is isolated in an open area and reclined in the brusher’s lap. The patient is then immobilized by an extra attendant while the brusher institutes proper oral care. If a child cannot be adequately immobilized by one person, then both parents and perhaps siblings may be required to complete the home dental care procedures.
5. The standing and resistive child are placed in front of the caregiver so that the adult can wrap his or her legs around the child to support the torso while using the hands to support the head and brush the teeth.^47^

Technique often recommended is the horizontal scrub method, because it is easy to perform and can yield good results. Electric toothbrushes have also been used effectively by children with disabilities. ^47^

In addition to head control, a common obstacle to oral cleansing and dental care is hand interference (i.e. patient grabs caregiver’s or dental provider’s hands). Stabilization of the patient’s elbows can contribute to oral cleansing, provides safety for the patient and caregiver and enables support personnel to restrict movement.^49^

## ANESTHESIA IN CEREBRAL PALSY PATIENTS

Children with CP present for surgery for a variety of reasons, ranging from general surgical conditions to particular interventions. These procedures are offered to a broad crosssection of patients and may be indicated for improvement in mobilization or ease of care, in keeping with the broad goals of managing children with CP.^50^

*Medical history*: It is essential to obtain an accurate medical history. An evaluation for pre-existing medical conditions must be performed before any therapeutic measures.

*Dental history*: The dental history is elicited from the aide or parents who brought the patient. What was done in the past and how it was done (with or without sedation/ general anesthesia) will help to give the practitioner a better understanding of how to manage the patient.^51^

*Criteria for dental care under general anesthesia*: Patients with special needs require the same level of assessment as regular patients. Specific documentation about presence of caries, nonrestorable teeth, impactions, significant calculus assists in planning the time for the scheduled care under general anesthesia.^52^

*Consent*: Consent forms for sedation can be given to the aide accompanying the patient or mailed directly to the legal guardian/parent if the legal guardian/parent will not be present on the day of sedation. Points to be noted include the following: How extensive is the dentistry that needs to be done? Can the patient’s treatment plan be completed in one to three 2-hour sedation visits or will the treatment take a longer amount of time? How manageable is the patient? Is the patient cooperative enough to get into the dental chair with a papoose board on his/her own or with gentle urging by the residency staff, or is the patient combative and does he/she pose a physical danger to you, your staff, or him/ herself? What is the medical and physical condition of the patient? Is the patient obese and unable to fit into a papoose board? Is the patient considered an ASA III patient and not a candidate for outpatient IV sedation?^51^

*Day of treatment*: The legal guardians are informed of the date of the sedation in advance and are asked to be available for a phone call on that morning. Informed consent is discussed, including proposed procedures and other options.

*Intravenous medications*: The drugs should be appropriate to the practitioner’s comfort level and match what is being done from a dental treatment and time perspective. Common categories of drugs used for sedation are benzodiazepines, narcotics, propofol and adjunct drugs, such as an antisialagogue.^51^

*Conscious sedation*: Nitrous oxide and oxygen through a nasal mask are other medications that help in sedating the patient. Many in the severe and profound levels of mental disability will not tolerate the mask on their faces first without IV medications. On many occasions, those who have the mild-to-moderate range of disability will let a nasal mask be placed first if it is shown to them and its function explained. If the nasal mask is tolerated first, nitrous oxide and oxygen can be used to allay any fear and anxiety involved with the insertion of the IV catheter. Oxygen will raise the oxygen saturation of the blood, one of the vital signs monitored through pulse oximetry, decreasing the likelihood of hypoxia.

Jensen TM^53^ found a significant reduction in the movements when the patient was given nitrous oxide (70% nitrous oxide, 30% pure oxygen).

*For radiographs*: If radiographs need to be taken, an easy way to accomplish this is with the Rinn X-ray system (Dentsply Rinn, Elgin, Illinois). The radiograph is placed in the patient’s mouth in the usual fashion. Tape used for securing the IV is placed in the midline of the patient’s neck, going vertically over the patients chin and to the side of the patient’s nose.

*Maintaining patent airway*: For several reasons, it is important during sedation to protect the patient’s airway. The patient being sedated is supine in the dental chair and is at risk for aspiration of dental filling materials, debris from preparation of the tooth, an extracted tooth, or calculus being scaled. Saliva can be produced in copious amounts and can fow backward, too, causing coughing. A third consideration is the use of water for cooling instruments. A throat shield should always be used to protect the airway when a rubber dam is not in use. It should be changed frequently as it gets wet or full of debris.^51^

Postoperative evaluation with the sedative medications used today, the patient will usually recover within a short time. When treating special care patients who might not be able to obey or respond to verbal commands, it is better to keep the patient in the chair wrapped in the papoose board until consciousness is fully restored and the patient can ambulate. Many patients are intolerant of having an IV or monitors attached once alert. Therefore, they should be removed beforehand. Written postoperative instructions are given, along with a written note of what treatment was accomplished, the dental recommendations for the next IV sedation appointment to continue treatment or for recall appointment, any medications prescribed, oral hygiene recommendations and dental diagnosis.^51^

Loyola-Rodriguez et al^54^ concluded that general anesthesia (GA) with sevofurane, propofol and conscious sedation is an excellent tool to provide dental treatment in CP patients without most of the major postoperative complications.

## CONCLUSION AND SUMMARY

The diagnosis of CP not always is straightforward, but an early diagnosis is important in terms of optimizing therapeutic interventions. The role of the pediatric healthcare provider is also to help families manage the ongoing health issues that may arise, and to give the families the confidence, they are doing all that they can and should do to help their child reach his or her potential. As oral health is increasingly recognized as a foundation for general health and wellness and a primary indicator for the success of dental treatment, caregivers for special need patients are an essential component of the oral health team and must become knowledgeable and competent in home oral health practice. Home oral health practice is a significant factor in dental care, general health, quality of life and controlling health care costs.
